# The Role of Glycogen Synthase Kinase-3 in the Regulation of Ribosome Biogenesis in Rat Soleus Muscle under Disuse Conditions

**DOI:** 10.3390/ijms23052751

**Published:** 2022-03-02

**Authors:** Sergey V. Rozhkov, Kristina A. Sharlo, Boris S. Shenkman, Timur M. Mirzoev

**Affiliations:** Myology Laboratory, Institute of Biomedical Problems RAS, 123007 Moscow, Russia; rozhkov.work@yandex.ru (S.V.R.); sharlokris@gmail.com (K.A.S.); bshenkman@mail.ru (B.S.S.)

**Keywords:** soleus muscle, hindlimb unloading, simulated microgravity, GSK-3, ribosome biogenesis, muscle protein synthesis, c-Myc, 18S rRNA, 28S rRNA

## Abstract

It is well-established that prolonged exposure to real or simulated microgravity/disuse conditions results in a significant reduction in the rate of muscle protein synthesis (PS) and loss of muscle mass. Muscle protein synthesis is largely dependent upon translational capacity (ribosome content), the regulation of which is poorly explored under conditions of mechanical unloading. Glycogen synthase kinase-3 (GSK-3) (a negative regulator of PS) is known to be activated in rat soleus muscle under unloading conditions. We hypothesized that inhibition of GSK-3 activity under disuse conditions (hindlimb suspension, HS) would reduce disuse-induced downregulation of ribosome biogenesis in rat soleus muscle. Wistar rats were randomly divided into four groups: (1) vivarium control (C), (2) vivarium control + daily injections (4 mg/kg) of AR-A014418 (GSK-3 inhibitor) for 7 days, (3) 7-day HS, (4) 7-day HS + daily injections (4 mg/kg) of AR-A014418. GSK-3beta and glycogen synthase 1 (GS-1) phosphorylation levels were measured by Western-blotting. The key markers of ribosome biogenesis were assessed via agarose gel-electrophoresis and RT-PCR. The rate of muscle PS was assessed by puromycin-based SUnSET method. As expected, 7-day HS resulted in a significant decrease in the inhibitory Ser9 GSK-3beta phosphorylation and an increase in GS-1 (Ser641) phosphorylation compared to the C group. Treatment of rats with GSK-3 inhibitor prevented HS-induced increase in GS1 (Ser641) phosphorylation, which was indicative of GSK-3 inhibition. Administration of GSK-3 inhibitor partly attenuated disuse-induced downregulation of c-Myc expression as well as decreases in the levels of 45S pre-rRNA and 18S + 28S rRNAs. These AR-A014418-induced alterations in the markers of ribosome biogenesis were paralleled with partial prevention of a decrease in the rate of muscle PS. Thus, inhibition of GSK-3 during 7-day HS is able to partially attenuate the reductions in translational capacity and the rate of PS in rat soleus muscle.

## 1. Introduction

Skeletal muscle disuse/mechanical unloading occurring during real or simulated microgravity, bedrest, limb immobilization, mechanical ventilation causes a significant reduction in muscle mass, force production and overall function [1,2,3,4,5]. Moreover, transient periods of muscle inactivity can accelerate losses of muscle and strength in older individuals with sarcopenia leading to a variety of negative health outcomes, including an increased risk for falls, fractures and the development of metabolic diseases [6]. Therefore, understanding the processes that contribute to disuse-induced muscle atrophy is an important area of research. Decreased rates of muscle protein synthesis (PS) and increased rates of muscle protein breakdown are considered to be the primary determinants of inactivity-induced muscle atrophy [7].

The rate of PS is largely dependent upon translational capacity (amount of ribosomes per unit tissue), the regulation of which is poorly explored under conditions of mechanical unloading/disuse. Ribosome biogenesis plays a key role in driving increased translational capacity in skeletal muscle following resistance training [8,9,10]. In contrast, ribosome biogenesis appears to be downregulated in both human and rat skeletal muscles under disuse/unloading conditions [11,12,13,14,15,16,17,18]. Ribosome biogenesis is a complex cellular process that involves synthesis of new ribosomes and requires the coordinated actions of the three types of RNA polymerase (RNA Pol I, II, and III) [19]. RNA Pol I is needed for the transcription of the polycistronic 47S pre-rRNA, which is subsequently processed to yield 18S, 5.8S, and 28S ribosomal RNAs (rRNAs) [20]. Genes that encode ribosomal proteins are transcribed by RNA Pol II, while transcription of 5S rRNA requires RNA Pol III [20]. It is worth noting that the synthesis of 47S pre-rRNA is considered to be a rate-limiting step in ribosome biogenesis [21,22,23]. Since the processing of primary 47S transcript is rapid [24], 47S pre-rRNA levels reflect the rate of rRNA synthesis at a particular time-point [21].

Glycogen synthase kinase 3 (GSK-3) is a multifaceted Ser/Thr protein kinase known to phosphorylate several hundred substrates [25], thereby regulating a wide range of intracellular processes including protein turnover in skeletal muscle [26]. GSK-3 exists in two isoforms, GSK-3α and GSK-3β, among which GSK-3β is considered to be the most expressed and active isoform within skeletal muscles [27]. GSK-3β can be involved in the negative control of translational capacity via regulation of β-catenin and transcription factor myelocytomatosis oncogene (c-Myc). c-Myc is known to stimulate transcription of rRNA genes by RNA Pol I [28,29]. Moreover, c-Myc regulates genes encoding ribosomal proteins and promotes transcriptional activity of RNA polymerase III [19]. Inhibition of GSK-3 results in an inability of GSK-3 to phosphorylate and inactivate β-catenin [19]. Unphosphorylated β-catenin is able to translocate to the nucleus and activate growth-control genes including c-Myc [30]. Thus, literature suggests that GSK-3 can be implicated in the negative regulation of ribosome biogenesis in skeletal muscles. Since disuse/mechanical unloading leads to a significant increase in GSK-3β activity in skeletal muscle (as assessed by a decrease in inhibitory Ser9 phosphorylation) [11,14,31,32,33], we hypothesized that GSK-3 may be involved in the regulation of ribosome biogenesis in the atrophying skeletal muscle. The aim of the present study was to elucidate whether inhibition of GSK-3 activity under disuse conditions (rat hindlimb suspension model, HS) would reduce downregulation of ribosome biogenesis in rat soleus muscle.

## 2. Results

### 2.1. Body Weight, Soleus Muscle Wet Weight and Soleus Weight-to-Body Weight Ratio

Mean body weight did not significantly differ between groups (Table 1). One-week HS resulted in a significant 27% decrease in rat soleus muscle wet weight and 25% reduction in soleus weight-to-body weight ratio compared to the C group (Table 1). Absolute and normalized soleus muscle mass in AR-A014418 treated unloaded rats (the 7HS + AR group) declined by 22% and 23%, respectively, relative to weight-bearing control rats (Table 1).

### 2.2. Effect of AR-A014418 on Cross-Sectional Area (CSA) of Soleus Muscle Fibers

**Figure 1:**
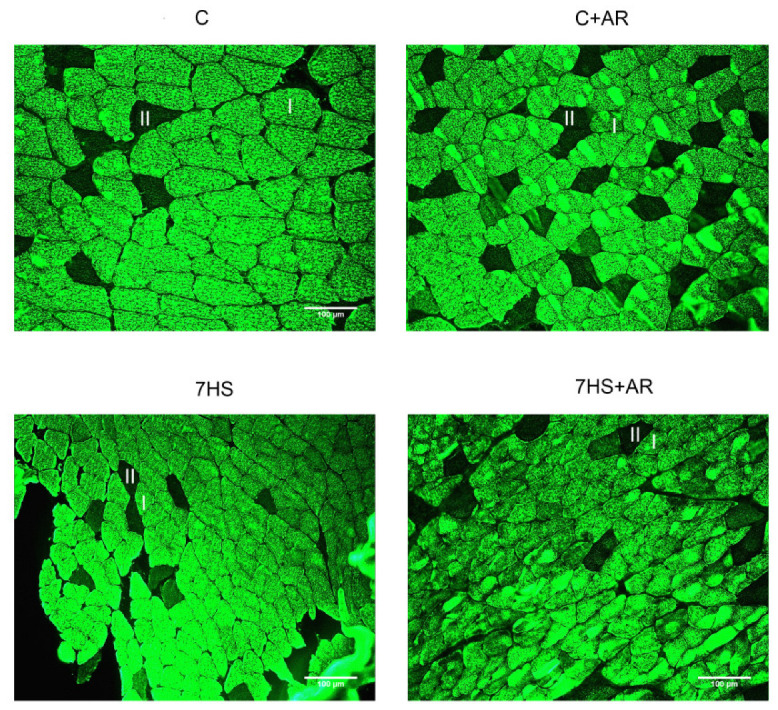
Representative images showing type I fibers (I, green) and type II fibers (II, dark) in rat soleus muscle. C, vivarium control group, C + AR, vivarium control rats + daily injections of GSK-3 inhibitor (AR-A014418) for 7 days, 7HS, hindlimb suspension for 7 days, 7HS + AR, 7-day hindlimb suspension + daily injections of GSK-3 inhibitor (AR-A014418). Scale bar, 100 μm.

As expected, one-week HS induced a significant reduction in the mean CSA of both slow-type (I) and fast-type (II) muscle fibers (Table 2, Figure 1). The mean CSA of slow-type fibers in the 7HS + AR group did not significantly differ from the C and C + AR groups. GSK-3 inhibition did not affect the mean CSA of fast-type fibers in rat soleus muscle during HS (Table 2, Figure 1).

### 2.3. Effect of AR-A014418 on GSK-3β Ser9 Phosphorylation, GS-1 Ser 641 Phosphorylation and β-Catenin Content

**Figure 2:**
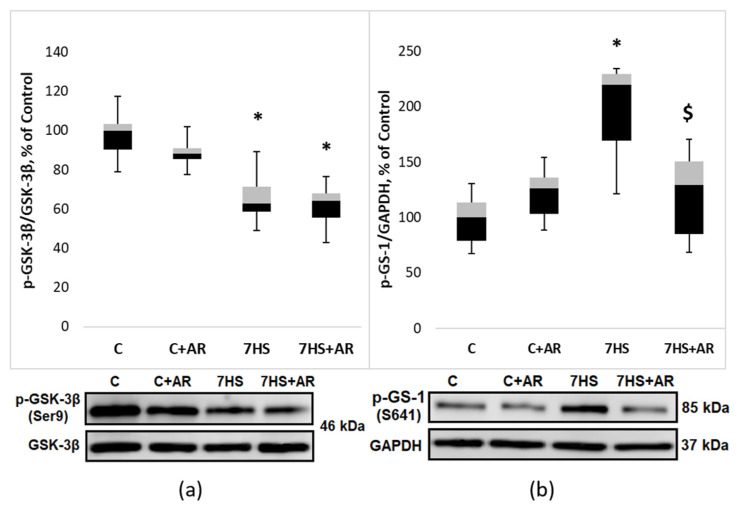
Effect of GSK-3 inhibition during 7-day unloading on GSK-3β Ser9 phosphorylation (**a**) and GS-1 Ser 641 phosphorylation (**b**) in rat soleus muscle. Data are presented as median, interquartile range, minimum and maximum values, expressed as % of control. C, vivarium control group, C + AR, vivarium control rats + daily injections of GSK-3 inhibitor (AR-A014418) for 7 days, 7HS, hindlimb suspension for 7 days, 7HS + AR, 7-day hindlimb suspension + daily injections of GSK-3 inhibitor (AR-A014418), *: *p* < 0.05 vs. C, $: *p* < 0.05 vs. 7HS.

**Figure 3:**
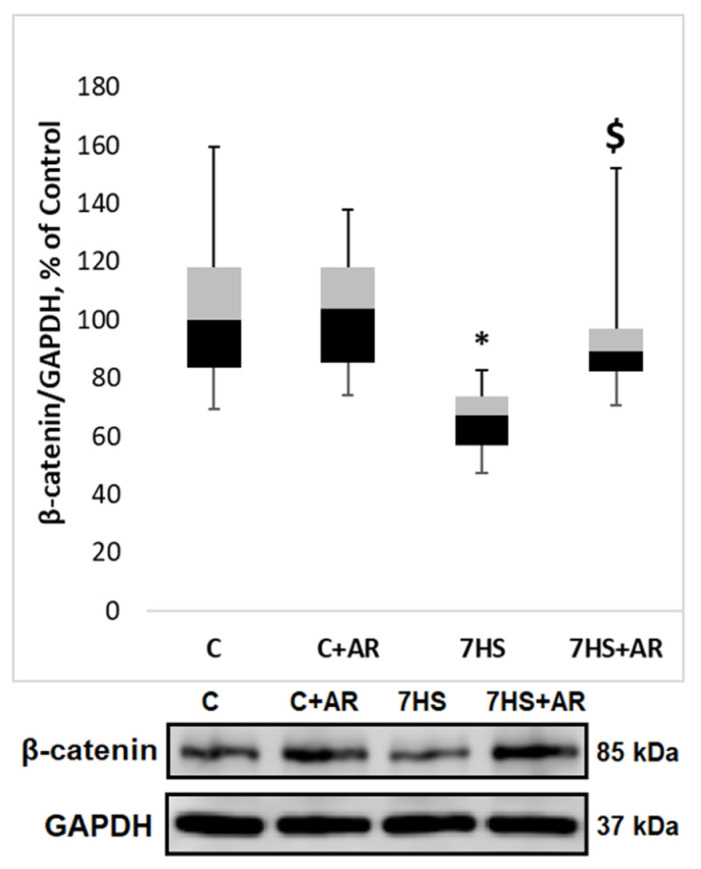
Effect of GSK-3 inhibition during 7-day unloading on β-catenin content in rat soleus muscle. Data are presented as median, interquartile range, minimum and maximum values, expressed as % of control. C, vivarium control group, C + AR, vivarium control rats + daily injections of GSK-3 inhibitor (AR-A014418) for 7 days, 7HS, hindlimb suspension for 7 days, 7HS + AR, 7-day hindlimb suspension + daily injections of GSK-3 inhibitor (AR-A014418), *: *p* < 0.05 vs. C, $: *p* < 0.05 vs. 7HS.

A period of 7 days of mechanical unloading resulted in a significant 37% decline in GSK-3β Ser9 phosphorylation (indicative of GSK-3β activation) in rat soleus muscle compared to control values (Figure 2a). Administration of GSK-3 inhibitor AR-A014418 during the unloading period did not significantly affect GSK-3β Ser9 phosphorylation versus the 7HS group (Figure 2a). The absence of the effect of AR-A014418 on GSK-3β Ser9 phosphorylation in the 7HS + AR group was expected since AR-A014418 inhibits GSK-3 in an ATP-competitive manner and does not affect posttranslational modifications (Ser phosphorylation). Therefore, we further assessed phosphorylation status of glycogen synthase 1 (GS-1), a key GSK-3 substrate. As expected, 7-day HS resulted in a significant 119% increase in GS-1 Ser 641 phosphorylation compared to the C group (Figure 2b). Treatment of HS rats with AR-A014418 resulted in a significant attenuation of GS-1 Ser 641 phosphorylation in rat soleus muscle (Figure 2b), which was indicative of a decrease in GSK-3 activity. A significant reduction in GSK-3 activity in the 7HS + AR group was also confirmed by assessing the content of another GSK-3 substate, β-catenin. GSK-3 is known to phosphorylate β-catenin leading to its ubiquitination and proteasomal degradation. In accordance with changes in GSK-3 activity (evidenced by GS-1 phosphorylation), β-catenin content in rat soleus muscle significantly declined in the 7HS group versus the C group and AR-A014418 treatment prevented an unloading-induced decrease in total β-catenin content in rat soleus muscle (Figure 3). Treatment of weight-bearing control rats with AR-A014418 for 7 days (C + AR group) had no effect on phosphorylation of GSK-3β and GS-1 (Figure 2) as well as β-catenin content (Figure 3) in rat soleus muscle.

### 2.4. Effect of AR-A014418 on the Key Markers of Ribosome Biogenesis in Rat Soleus Muscle

Total RNA content (normalized to tissue weight, μg/mg) in rat soleus muscle following 7-day HS significantly declined by 44% compared to the C group (Table 3). Administration of AR-A014418 during 7-day HS resulted in a significant attenuation of total RNA decline as shown in Table 3 below.

**Figure 4:**
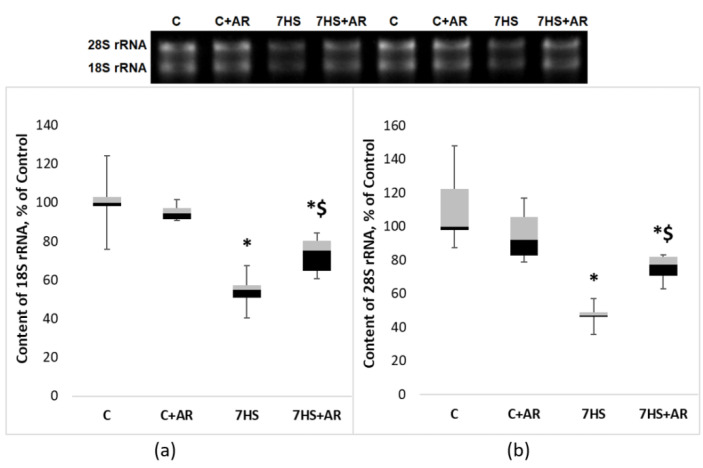
Effect of GSK-3 inhibition during 7-day unloading on the amount of 18S rRNA (**a**) and 28S rRNA (**b**) in rat soleus muscle. Data are presented as median, interquartile range, minimum and maximum values, expressed as % of control. C, vivarium control group, C + AR, vivarium control rats + daily injections of GSK-3 inhibitor (AR-A014418) for 7 days, 7HS, hindlimb suspension for 7 days, 7HS + AR, 7-day hindlimb suspension + daily injections of GSK-3 inhibitor (AR-A014418), *: *p* < 0.05 vs. C, $: *p* < 0.05 vs. 7HS.

One-week mechanical unloading resulted in a significant decrease in 18S rRNA content (−45%) and 28S rRNA content (−53%) relative to the C group (Figure 4). Treatment of weight-bearing control rats with AR-A014418 for 7 days (C + AR group) did not affect the content of 18S rRNA and 28S rRNA (Figure 4). However, GSK-3 inhibition during 7-day HS induced a significant attenuation of a decline in 18S and 28S rRNAs in rat soleus muscle (Figure 4).

**Figure 5:**
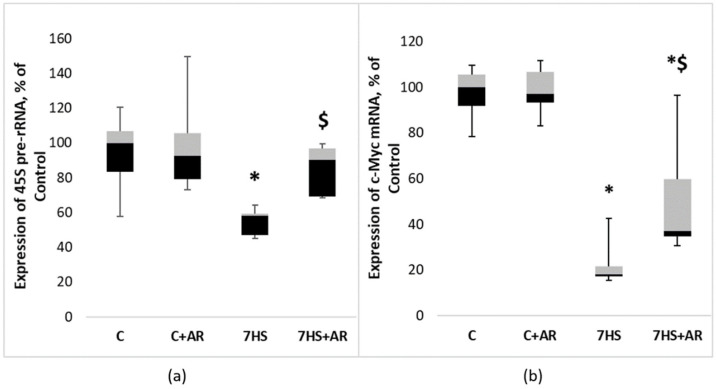
Effect of GSK-3 inhibition during 7-day unloading on the expression of 45S pre-rRNA (**a**) and c-Myc (**b**) in rat soleus muscle. Data are presented as median, interquartile range, minimum and maximum values, expressed as % of control. C, vivarium control group, C + AR, vivarium control rats + daily injections of GSK-3 inhibitor (AR-A014418) for 7 days, 7HS, hindlimb suspension for 7 days, 7HS + AR, 7-day hindlimb suspension + daily injections of GSK-3 inhibitor (AR-A014418), *: *p* < 0.05 vs. C, $: *p* < 0.05 vs. 7HS.

Mature 18S and 28S rRNAs are generated by a complex processing of 45S pre-rRNA. As shown in Figure 5a, 7 days of HS resulted in a significant reduction in 45S pre-rRNA expression in rat soleus muscle compared to the C group. Inhibition of GSK-3 activity prevented HS-induced decline in the expression levels of 45S pre-rRNA (Figure 5a). Expression levels of the key regulator of ribosome biogenesis, proto-oncogene c-Myc, profoundly declined (−82%) after 7-day HS, however, GSK-3 inhibition during 7-day HS partly mitigated c-Myc downregulation in rat soleus muscle (Figure 5b).

### 2.5. Effect of AR-A014418 on the Phosphorylation Status of Ribosomal Protein S6 (rpS6) and Translation Initiation Factor 4E-Binding Protein 1 (4E-BP1)

**Figure 6:**
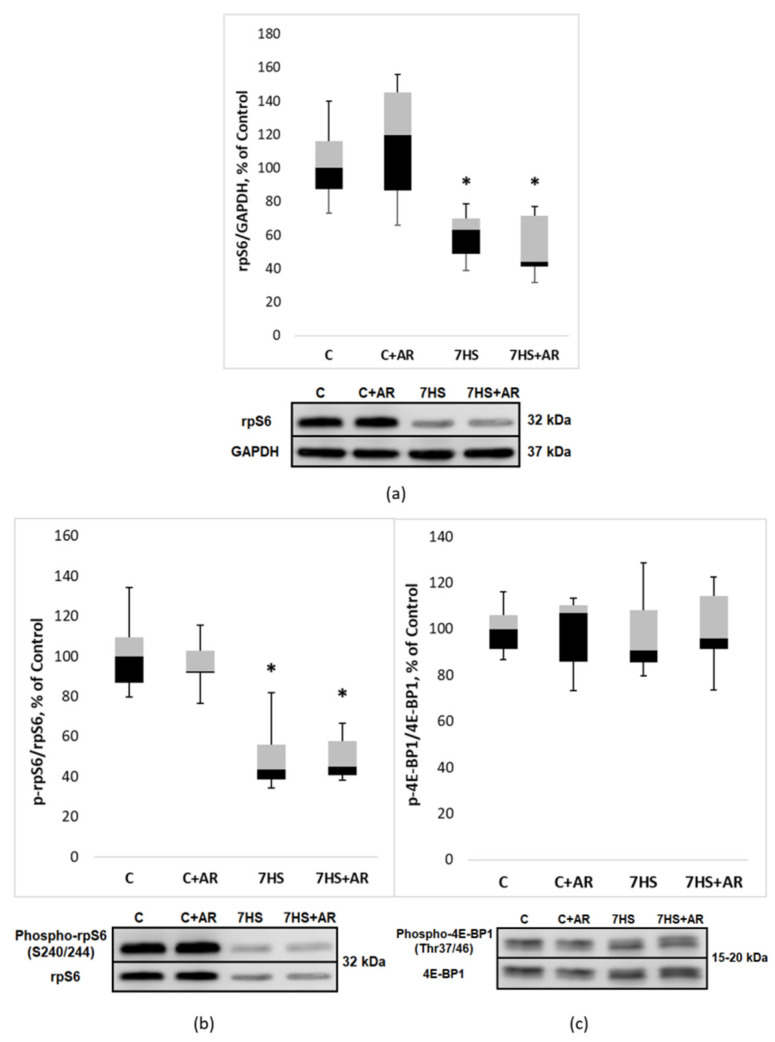
Effect of GSK-3 inhibition during 7-day unloading on rpS6 abundance (**a**) and phosphorylation status of rpS6 (**b**) and 4E-BP1 (**c**) in rat soleus muscle. Data are presented as median, interquartile range, minimum and maximum values, expressed as % of control. C, vivarium control group, C + AR, vivarium control rats + daily injections of GSK-3 inhibitor (AR-A014418) for 7 days, 7HS, hindlimb suspension for 7 days, 7HS + AR, 7-day hindlimb suspension + daily injections of GSK-3 inhibitor (AR-A014418), *: *p* < 0.05 vs. C.

One-week mechanical unloading resulted in a significant decline in both total rpS6 content and phospho-rpS6/total rpS6 ratio compared to the C group (Figure 6a,b). Treatment of rats with AR-A014418 during HS affected neither rpS6 abundance nor rpS6 phosphorylation (Figure 6a,b). As shown in Figure 6c, no significant differences were found between the groups in terms of 4E-BP1 phosphorylation.

### 2.6. Effect of AR-A014418 on the Rate of Protein Synthesis in Rat Soleus Muscle

**Figure 7:**
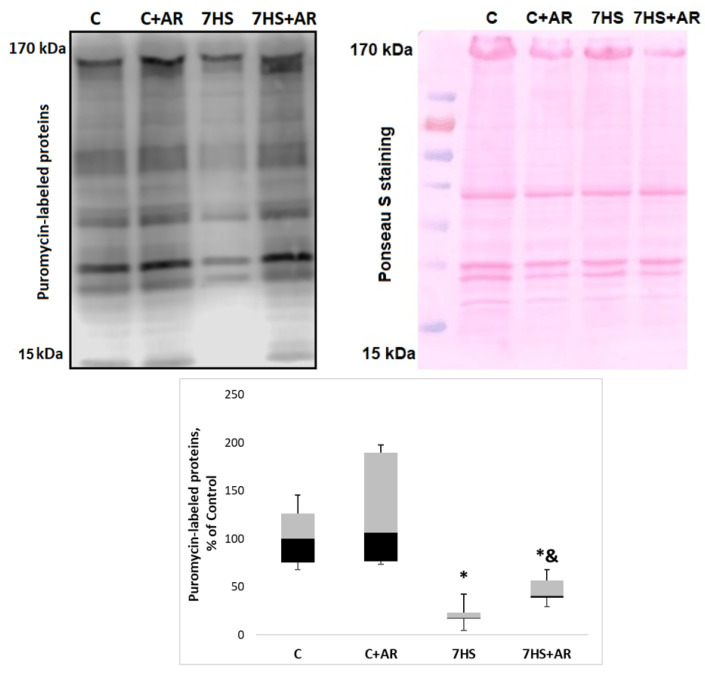
Effect of GSK-3 inhibition during 7-day unloading on the rate of protein synthesis in rat soleus muscle. Data are presented as median, interquartile range, minimum and maximum values, expressed as % of control. C, vivarium control group, C + AR, vivarium control rats + daily injections of GSK-3 inhibitor (AR-A014418) for 7 days, 7HS, hindlimb suspension for 7 days, 7HS + AR, 7-day hindlimb suspension + daily injections of GSK-3 inhibitor (AR-A014418), *: *p* < 0.05 vs. C, &: *p* < 0.05 vs. 7HS.

Mechanical unloading for 7 days induced a profound decline in the rate of protein synthesis (−82%) in rat soleus muscle relative to the weight-bearing control rats (Figure 7). Treatment of the unloaded rats with AR-A014418 (GSK-3 inhibitor) resulted in partial attenuation of a reduction in the protein synthesis rates in rat soleus muscle as shown in Figure 7. The rate of protein synthesis in the 7HS + AR group was 16% higher than that in the 7HS group (Figure 7). Treatment of weight-bearing control rats with AR-A014418 for 7 days (C + AR group) did not affect the rate of muscle protein synthesis (Figure 7).

## 3. Discussion

In this study, we questioned if GSK-3 inhibition by AR-A014418 treatment would impact translational capacity (i.e., ribosome biogenesis) in rat postural muscle during mechanical unloading (7-day HS). The present study shows that GSK-3 inhibition ameliorates HS-associated reduction in ribosome biogenesis in rat soleus muscle. In particular, daily treatment of rats with GSK-3 inhibitor AR-A014418 during 7-day HS attenuated a decrease in such markers of ribosome biogenesis as c-Myc, 45S pre-rRNA, total RNA and 18S + 28S rRNAs in rat soleus muscle. Moreover, GSK-3 inhibition partly rescued a HS-induced decline in the global rates of PS in rat soleus muscle, as evidenced by puromycin-based SUnSET method.

One-week HS resulted in a significant decrease in GSK-3β (Ser 9) phosphorylation in rat soleus muscle, which was indicative of the enhancement of GSK-3β enzymatic activity. GSK-3β activation after 7-day HS was confirmed by a significant increase in GS-1 Ser 641 phosphorylation and downregulation of β-catenin content, key substrates of GSK-3β. The finding of decreased Ser 9 GSK-3β phosphorylation in rodent soleus muscle during the first week of HS is consistent with a number of previously published studies [11,14,18,31,34].

Treatment of rats with AR-A014418 (a selective GSK-3 inhibitor acting in an ATP competitive manner) during 7-day unloading prevented an increase in GS-1 (Ser641) phosphorylation and β-catenin degradation, which was indicative of GSK-3 inhibition. One possible mechanism connecting GSK-3 activity and ribosome biogenesis may involve a repression of RNA polymerase I transcription by GSK-3β. In epithelial cells transformed with oncogenic RAS, Vincent et al. (2008) have demonstrated that GSK-3β associates with promoter and coding region of the rDNA and GSK-3β inhibition is able to upregulate 18S and 28S rRNA synthesis [35]. Furthermore, given that GSK-3 can be involved in the control of translational capacity via regulation of β-catenin (phosphorylation of β-catenin by GSK-3 primes it for ubiquitination and subsequent destruction by the proteasome [36]) and the latter is known to activate the expression of c-Myc, a key regulator of ribosome biogenesis [30], GSK-3/β-catenin/c-Myc pathway may represent another possible mechanism linking GSK-3 activity and ribosome biogenesis in skeletal muscle. Indeed, in the present study, the HS-induced reduction in c-Myc mRNA expression was partly attenuated by treatment of rats with GSK-3 inhibitor (AR-A014418). A significant unloading-induced downregulation of c-Myc in rat soleus muscle is line with our previously published data on alterations in c-Myc expression following 3- or 7-day HS [11,16,18]. Since c-Myc plays a key role in the regulation of ribosome biogenesis via activation of Pol I transcription [28], Pol III transcription [37] and promotion of the opening of the chromatin structure near rDNA loci through the acetylation of histones [28,29], we hypothesized that inhibition of the increased GSK-3 activity due to 7-day HS would prevent or, at least, partly attenuate reductions in translational capacity and thereby contribute to the maintenance of protein synthesis rates in rat soleus muscle during unloading conditions. In agreement with this hypothesis, we observed that GSK-3 inhibition during 7-day HS partially rescued a decline in ribosome biogenesis, as assessed by such markers as 45S pre-rRNA, total RNA (rRNAs make up about 80% of total RNA) and 18S + 28S rRNAs (components of the ribosomal small (40S) and large (60S) subunits, respectively). Furthermore, GSK-3 inhibition-induced maintenance of ribosome biogenesis during unloading conditions correlated with partial attenuation of the rate of muscle protein synthesis. Of note, a growing body of evidence suggests that GSK-3 activity in skeletal muscles (and, hence, β-catenin/c-Myc pathway) is largely dependent on endogenous production of nitric oxide (NO), which is mediated by NO-synthase [11,34,38,39]. It was shown that HS can induce a significant reduction in NO levels in rat soleus muscle, which correlates with a decrease in inhibitory phosphorylation of GSK3-beta at Ser9 (i.e., increased GSK-3beta activity) [40]. Moreover, in C2C12 myotubes it was demonstrated that NO-related GSK3-beta Ser9 phosphorylation can be mediated via soluble guanylate cyclase/cyclic guanosine monophosphate (cGMP)/cGMP-dependent kinase pathway [39].

It is interesting to note that c-Myc is able to enhance protein synthesis during tumorigenesis by activating mechanistic target of rapamycin complex 1 (mTORC1)-dependent phosphorylation of 4E-BP1 [41], a key protein involved in the regulation of mRNA translation initiation. Furthermore, c-Myc was shown to be implicated in the transcriptional regulation of a number of ribosomal proteins (including rpS6) and translation initiation factors [42,43]. However, while treatment of rats with GSK-3 inhibitor partially rescued the unloading-induced decline in c-Myc expression in the present study, no changes in rpS6 abundance/phosphorylation or 4E-BP1 phosphorylation were found in the 7HS+AR group vs. the 7HS group. These results suggest that GSK-3- and Myc-related regulation of ribosome biogenesis in rat soleus muscle during mechanical unloading is presumably mTORC1-independent. Our c-Myc results are in agreement with a study by West et al. (2016) showing a decrease in insulin-like growth factor-induced protein synthesis in C2C12 myotubes in response to c-Myc inhibition while 4E-BP1 and rpS6 phosphorylation levels remain high [44].

The findings of the present study pertaining to disuse-induced changes in the markers of translational capacity and muscle protein synthesis are in good agreement with previous reports involving rodents as well as humans. Specifically, a substantial decrease in total RNA content observed in our study following 7-day HS is corroborated by a recent study by Figueiredo et al. (2021) in which middle-aged men (50 ± 3.54 years old) underwent 2 weeks of unilateral limb immobilization and male Brown Norway/F344 rats (10 months of age) were subjected to 1- and 7-day HS [15]. Furthermore, a significant decrease in total RNA in human vastus lateralis muscle was previously shown following 10 days of lower limb unloading [17] as well as 5 weeks of unilateral lower limb suspension [45]. Overall, a large number of studies consistently showed a significant reduction in total RNA in rodent skeletal muscles in response to unloading/disuse conditions [12,13,14,16,46]. As expected, similar pattern of alterations was observed for the key components of the small and large ribosomal subunits, 18S rRNA and 28S rRNA, respectively. Our findings concerning effects of HS on the amount of 18S + 28S rRNAs in rat soleus muscle are in accord with earlier published data [13,16]. It is known that the formation of 18S and 28S rRNAs is a result of 45S pre-mRNA processing [19]. In the present study, 7-day unloading induced a significant decrease in 45S pre-rRNA expression in rat soleus, which was attenuated by GSK-3 inhibition. Unloading-induced downregulation of 45S pre-rRNA in rat soleus muscle conforms well to recently published reports on the effects of hindlimb unloading of various durations on ribosome biogenesis in skeletal muscles [15,16].

It is noteworthy that although inhibition of GSK-3 during 7-day unloading partly attenuated downregulation of the markers of translational capacity and the rate of protein synthesis, decreased soleus muscle mass was not rescued, despite partial protection of the CSA of slow-type muscle fibers. One possible explanation of this result is that an increase in the size of exclusively slow-type muscle fibers in the 7HS + AR group vs. the 7HS group was not enough to attenuate a decrease in the whole muscle mass.

## 4. Materials and Methods

### 4.1. Experimental Design

Male Wistar rats (2.5 months of age, 200 ± 10 g, obtained from the Nursery for Laboratory Animals of the Institute of Bioorganic Chemistry of the RAS, Pushchino, Moscow region) were randomly divided into the following 4 groups (n = 8 per group): (1) vivarium control (i.e., weight bearing conditions) (C), (2) vivarium control plus daily intraperitoneal (i.p.) injections of GSK-3 inhibitor (AR-A014418) for 7 days (C + AR), (3) 7-day hindlimb suspension (i.e., unloading/disuse conditions) (7HS), and (4) 7-day hindlimb suspension plus daily i.p. injections of GSK-3 inhibitor (AR-A014418) (7HS + AR). Mechanical unloading/disuse was performed via tail suspension (hindlimb suspension), as previously described by Morey-Holton and Globus (2002) [47]. AR-A014418 (#A3184; APExBIO, Houston, TX, USA) was administered once a day (each injection contained 4 mg/kg in saline + 1% DMSO). The applied dose of AR-A014418 was selected based on our previous research [34]. Rats from the C and 7HS groups received an equivalent volume of saline + 1% DMSO without AR-A014418. The rats were anesthetized with an i.p. injection of tribromoethanol (240 mg/kg, #T48402; Sigma-Aldrich, St. Louis, MO, USA) and soleus muscles were dissected, weighed and frozen in liquid nitrogen until further analysis. Upon completion of the experiment, the rats were sacrificed by i.p. injection of tribromoethanol overdose (750 mg/kg, #T48402; Sigma-Aldrich, St. Louis, MO, USA) followed by cervical dislocation.

### 4.2. Determination of the CSA of Muscle Fibers

Cross-sections from the mid-belly of the muscles were cut at 10 μm in a cryostat (Leica Microsystems, Mannheim, Germany) maintained at −20 °C. The sections were then warmed to room temperature for 15 min and rehydrated by incubating in PBS for 20 min. The rehydrated sections were then incubated for 1 h at 37 °C with primary antibodies against slow myosin heavy chains (MyHC I (slow), 1:400, M8421, Merck, Darmstadt, Germany). After washing with PBS, the sections were incubated with the appropriate fluorophore-conjugated secondary antibodies (goat anti-mouse secondary antibody, Alexa Fluor 488, 1:500, A-11001, Thermo Fisher Scientific, Waltham, MA, USA) for 40 min in the dark at room temperature. After washing in PBS, the stained sections were mounted using the mounting medium for microscopic analysis. The sections were examined and photographed using a Leica Q500MC fluorescence microscope with an integrated digital camera (TCM 300F, Leica, Germany), 20× magnification. Image analysis was performed using ImageJ 1.52a software. At least 150 fibers were analyzed in each muscle sample.

### 4.3. Protein Synthesis Measurements

In vivo global rates of muscle protein synthesis were detected using a nonradioactive puromycin-based surface sensing of translation (SUnSET) method as previously described [48]. Puromycin dihydrochloride (Enzo Life Sciences, catalog no. BML-GR312-0250, Lausen, Switzerland) was dissolved in sterile saline and delivered by i.p. injection (0.04 μmol/g) 30 min before muscle collection. Puromycin-labeled peptides, reflecting the rate of protein synthesis, were analyzed by Western blotting.

### 4.4. Western Blotting

Western blot analysis was performed as described in our previous studies [49,50,51]. In brief, frozen muscles were homogenized in RIPA Lysis Buffer System (sc-24948, Santa Cruz Biotechnology, Santa Cruz Biotechnology, Dallas, TX, USA) and following centrifugation the supernatant was collected and protein concentrations were quantified using Bradford protein assay. Thirty micrograms of protein were subjected to SDS-PAGE and then transferred to nitrocellulose membrane (Bio-Rad Laboratories, Hercules, CA, USA). After blocking in 4% nonfat milk, the membranes were probed with primary antibodies (overnight at 4 °C) against p-GSK-3β (Ser 9) (1:1000, Cell Signaling Technology, USA, #9322), GSK-3β (1:1000, Cell Signaling Technology, Danvers, MA, USA, #12456), p-GS-1 (Ser 641) (1:10,000, #ab81230, Abcam, Cambridge, UK), puromycin (1:2000, # MABE343, Merck, Billerica, MA, USA), β-catenin (1:1000, #ab16051, Abcam, Cambridge, UK), p-4E-BP1 (Thr37/46) (1:1000, Cell Signaling Technology, Danvers, MA, USA, #2855), 4E-BP-1 (1:2000, Cell Signaling Technology, Danvers, MA, USA, #9452), p-rpS6 (S240/244) (1:2000, Cell Signaling Technology, Danvers, MA, USA, #5364), rpS6 (1:2000, Cell Signaling Technology, Danvers, MA, USA, #2217) and GAPDH (1:10,000, Applied Biological Materials Inc., Richmond, BC, Canada, no. G041). After that, the membranes were incubated for 1 h at room temperature with horseradish peroxidase-conjugated secondary antibodies to rabbit immunoglobulins (1:30000, Santa Cruz Biotechnology, Dallas, TX, USA, sc-2004). For detection of puromycin-labeled proteins secondary goat anti-mouse IgG (H + L)-HRP conjugate antibodies (1:35000; Bio-Rad Laboratories, Hercules, CA, USA, #1706516) were used. The protein bands were quantified using C-DiGit Blot Scanner (LI-COR Biotechnology, Lincoln, NE, USA) and Image Studio Digits software. For protein synthesis detection, the measurements of the chemiluminescent signals were performed by determining the density of each whole lane with the entire molecular weight range of puromycin-labeled peptides. GAPDH protein expression or total protein stain (Ponceau S) was used for normalization of Western blots.

### 4.5. RNA Isolation and Agarose Gel Electrophoresis

Frozen muscle tissue was cut using Leica CM 1900 cryostat (Mannheim, Germany) and weighed on electronic laboratory balance. Total RNA was extracted from muscle samples using RNeasy Micro Kit (Qiagen, Hilden, Germany) according to the manufacturer’s instructions. The concentration of isolated RNA was determined with a NanoPhotometer N50 Touch (IMPLEN, T50883, Munich, Germany,) at 260 nm. The amount of total RNA was calculated by multiplying the RNA concentration by the total volume of RNA solution. This value was divided by the tissue sample weight to obtain μg of total RNA per mg muscle tissue sample used for RNA isolation. RNA samples from equivalent amounts of tissue were run on gels in order to assess the content of 18S and 28S rRNAs. RNA quality of purification was evaluated according to 260/280 and 260/230 ratios. All RNA samples were mixed with equal value of denaturing buffer (Thermo Fisher Scientific Baltics, Vilnius, Lithuania, R0641) and heated for 10 min at 70 °C. The electrophoresis was performed in 1.2% agarose gel with ethidium bromide staining in TBE buffer (89 mM Tris, 89 mM boric acid, 2 mM EDTA, pH 8.0) at 10 V/cm. RiboRuler markers (Thermo Fisher Scientific Baltics, Vilnius, Lithuania, SM1821) were used for RNA molecular weight analysis. The measurements of the 28S and 18S rRNA were performed by Gel Doc EZ Imager (Bio-Rad Laboratories, Hercules, CA, USA).

### 4.6. Real-Time RT–PCR Analysis

To perform reverse transcription and real-time PCR, we determined raw RNA concentration, after which we selected from each sample a volume containing 1 µg of RNA. For reverse transcription to be provided, 1 μg RNA, oligo(dT)15, random hexamers d(N)6, dNTPs, RNase inhibitor, and Moloney Murine LeukemiaVirus (M-MLV) (Sintol, Moscow, Russia) reverse transcriptase (60 min at 37 °C) were used. For each target RNA, 1 μL cDNA was amplified in a 25 μL SYBR Green PCR reaction containing Quantitect SYBR Green Master Mix (Syntol, Moscow, Russia) and 10 μM of each primer: c-Myc-forward 5′-ttgatggggatgaccctgac-3′, c-Myc-reverse 5′-ctcgcccaaatcctgtacct-3′; 45S Pre-rRNA-forward 5′-gcctgtcactttcctccctg-3′, 45S Pre-rRNA-reverse 5′-gccgaaataaggtggccctc-3′; RPL19-forward 5′-gtacccttcctcttccctatgc-3′, RPL19-reverse 5′-caatgccaactctcgtcaucag-3′. The annealing temperature was based on the PCR primers’ optimal annealing temperature. The amplification was monitored in a real time using the iQ5 multicolor real-time PCR detection system (Bio-Rad Laboratories, Hercules, CA, USA). The identification of the PCR products was confirmed by melting-curve analysis after amplification. Quantification of the target genes was performed by using relative expression software tool (REST) software (v.2.0.12 Qiagen, Germany) which is based on an efficiency corrected mathematical model for data analysis as described by Pfaffl et al. (2002) [52]. In brief, the REST program computes the relative expression ratios on the basis of group means for target gene against reference gene and checks the group ratio results for significance. RPL19 was chosen as reference gene because its expression under unloading conditions was unchanged.

### 4.7. Statistical Analysis

Body/muscle weight, muscle fiber CSA and total RNA data are shown as mean ± SEM. Western blot, rRNA and mRNA data are shown as median and interquartile range (0.25–0.75) ± the minimum and the maximum. Sample medians in the boxplots are expressed as % of the control group. SigmaPlot 12.5 software package was used for statistical analysis. Since the normal distribution of the sample was not confirmed in all cases, a nonparametric Kruskal–Wallis test with Dunn’s multiple range test was applied. A *p* value less than 0.05 was regarded as statistically significant.

## 5. Conclusions

In conclusion, the present work revealed that GSK-3 activity during 7-day mechanical unloading/disuse can be involved in the regulation of ribosome biogenesis in rat postural soleus muscle. Inhibition of GSK-3 under unloading conditions partially attenuated a reduction in both ribosome biogenesis markers and the rate of muscle protein synthesis.

## Figures and Tables

**Table 1:** Changes in body weight, soleus wet weight and soleus weight-to-body weight ratio.

Groups	Body Weight, g	Soleus Wet Weight, mg	Soleus Weight-to-Body Weight Ratio, mg/g
C	230 ± 10	95 ± 5	0.43 ± 0.01
C + AR	250 ± 8	114 + 4	0.45 ± 0.02
7HS	215 ± 5	70 ± 3 *	0.32 ± 0.01 *
7HS + AR	225 ± 16	74 ± 5 *	0.33 ± 0.02 *

Values are means ± SEM, expressed as % of control. C, vivarium control group, C + AR, vivarium control rats + daily injections of GSK-3 inhibitor (AR-A014418) for 7 days, 7HS, hindlimb suspension for 7 days, 7HS + AR, 7-day hindlimb suspension + daily injections of GSK-3 inhibitor (AR-A014418), *: *p* < 0.05 vs. C.

**Table 2:** Changes in the mean CSA of slow-type (I) and fast-type (II) soleus muscle fibers.

Groups	CSA of Type I Fibers, μm^2^	CSA of Type II Fibers, μm^2^
C	3648 ± 244	2918 ± 68
C + AR	3300 ± 301	3049 ± 184
7HS	2182 ± 92 *	2130 ± 229 *
7HS + AR	2831 ± 414	1951 ± 163 *

Values are means ± SEM. C, vivarium control group, C + AR, vivarium control rats + daily injections of GSK-3 inhibitor (AR-A014418) for 7 days, 7HS, hindlimb suspension for 7 days, 7HS + AR, 7-day hindlimb suspension + daily injections of GSK-3 inhibitor (AR-A014418), *: *p* < 0.05 vs. C.

**Table 3:** Total RNA content in rat soleus muscle normalized per mg of muscle tissue.

Groups	Total RNA Content, % of Control
C	100 ± 5
C + AR	95 ± 3
7HS	56 ± 4 *
7HS + AR	88 ± 2 **^$^**

Values are means ± SEM. C, vivarium control group, C + AR, vivarium control rats + daily injections of GSK-3 inhibitor (AR-A014418) for 7 days, 7HS, hindlimb suspension for 7 days, 7HS + AR, 7-day hindlimb suspension + daily injections of GSK-3 inhibitor (AR-A014418), *: *p* < 0.05 vs. C, $: *p* < 0.05 vs. 7HS.

## Data Availability

The data presented in the study are available upon reasonable request from the corresponding author.
